# Comparative Assessment of the Effects of Climate Change on Heat- and Cold-Related Mortality in the United Kingdom and Australia

**DOI:** 10.1289/ehp.1307524

**Published:** 2014-09-15

**Authors:** Sotiris Vardoulakis, Keith Dear, Shakoor Hajat, Clare Heaviside, Bernd Eggen, Anthony J. McMichael

**Affiliations:** 1 Centre for Radiation, Chemical and Environmental Hazards, Public Health England, Chilton, United Kingdom; 2 National Centre for Epidemiology and Population Health, Australian National University, Canberra, Australia; 3 Department of Social and Environmental Health Research, London School of Hygiene and Tropical Medicine, London, United Kingdom; 4 Duke Global Health Institute, Duke University, Durham, North Carolina, USA

## Abstract

Background: High and low ambient temperatures are associated with increased mortality in temperate and subtropical climates. Temperature-related mortality patterns are expected to change throughout this century because of climate change.

Objectives: We compared mortality associated with heat and cold in UK regions and Australian cities for current and projected climates and populations.

Methods: Time-series regression analyses were carried out on daily mortality in relation to ambient temperatures for UK regions and Australian cities to estimate relative risk functions for heat and cold and variations in risk parameters by age. Excess deaths due to heat and cold were estimated for future climates.

Results: In UK regions, cold-related mortality currently accounts for more than one order of magnitude more deaths than heat-related mortality (around 61 and 3 deaths per 100,000 population per year, respectively). In Australian cities, approximately 33 and 2 deaths per 100,000 population are associated every year with cold and heat, respectively. Although cold-related mortality is projected to decrease due to climate change to approximately 42 and 19 deaths per 100,000 population per year in UK regions and Australian cities, heat-related mortality is projected to increase to around 9 and 8 deaths per 100,000 population per year, respectively, by the 2080s, assuming no changes in susceptibility and structure of the population.

Conclusions: Projected changes in climate are likely to lead to an increase in heat-related mortality in the United Kingdom and Australia over this century, but also to a decrease in cold-related deaths. Future temperature-related mortality will be amplified by aging populations. Health protection from hot weather will become increasingly necessary in both countries, while protection from cold weather will be still needed.

Citation: Vardoulakis S, Dear K, Hajat S, Heaviside C, Eggen B, McMichael AJ. 2014. Comparative assessment of the effects of climate change on heat- and cold-related mortality in the United Kingdom and Australia. Environ Health Perspect 122:1285–1292; http://dx.doi.org/10.1289/ehp.1307524

## Introduction

Scientific consensus indicates that anthropogenic climate change is likely to cause a range of direct and indirect effects on human health in developed and developing countries (Intergovernmental Panel on Climate Change 2014). These effects include an increase in heat-related mortality and morbidity in many parts of the world (Haines et al. 2006; McMichael et al. 2012). In the United Kingdom and Australia, annual and seasonal mean temperatures are generally projected to increase significantly over the 21st century, although these increases will not be geographically homogeneous [Australian Bureau of Meteorology (BoM) 2014; Jenkins et al. 2009]. The frequency of hot days (e.g., daily mean Central England Temperature > 20^o^C) has also substantially increased since 1900 and is likely to increase further in the future (Vardoulakis and Heaviside 2012). Similar temperature patterns have been observed in other regions in Europe and North America (Meehl and Tebaldi 2004).

Heat-related mortality is a matter of great public health concern, especially in the context of climate change (Huang et al. 2011). Climate change is expected to exacerbate the health impacts of heat through rising temperatures and higher frequency and severity of heat waves (Fischer and Schar 2010; Nitschke et al. 2011; Tong et al. 2010). Climate models also predict that extreme cold weather events are still likely to occur over European continental areas and other middle- and high-latitude regions under 21st-century warming scenarios (Kodra et al. 2011).

The magnitude of climate change–related impacts will vary substantially between countries and population groups. For example, the elderly are much more vulnerable to heat and cold than are younger age groups (Hajat et al. 2007, 2014). Heat- and cold-related health risks can also vary considerably across or within countries (Analitis et al. 2008; McMichael et al. 2008). Comparing health impacts in two or more countries, regions, or cities with different climatic or socioeconomic profiles can provide insights into risk factors (Gosling et al. 2009a; McMichael et al. 2008). There is evidence showing lower temperature thresholds for heat-related effects in “cooler” cities exhibiting lower mean summer temperatures than in “warmer” cities exhibiting higher temperatures (Gosling et al. 2009a). This suggests that populations can acclimatize and adapt to a warmer climate to some extent through physiological, spontaneous behavioral (e.g., wearing lighter clothes) and planned adaptation (e.g., thermal insulation, passive cooling, and ventilation in buildings, and planting of trees in cities) (O’Neill et al. 2009).

The scale and the pace of this adaptation are likely to depend on many location-specific parameters (e.g., built environment characteristics). Studies assessing future health impacts of climate change have often considered changes in mean temperature in the absence of any physiological, behavioral, or planned adaptation of the population to higher temperatures (Gosling et al. 2009a; Kinney et al. 2008). Currently, there is no standard method to account for the effect of acclimatization and adaptation to changing thermal conditions, which limits the predictive capacity of health impact assessment techniques (Hajat et al. 2014). A range of methods have been proposed for accounting for this effect, such as the analog cities (i.e., cities with different average temperatures but similar sociodemographic characteristics) and analog years (i.e., hottest years in past records) approaches (Gosling et al. 2009a; Huang et al. 2011; Kinney et al. 2008).

Although several studies have reported associations between ambient temperatures and mortality (Armstrong et al. 2011; Barnett et al. 2012; Basu 2009; Basu and Samet 2002; Gosling et al. 2009b; Hajat et al. 2007), there is less evidence on the likely future impacts of climate change on temperature related mortality (Christidis et al. 2010; Huang et al. 2011; Kinney et al. 2008). We have addressed this gap by estimating the direct effects of temperature exposure on public health in the United Kingdom and Australia over the 21st century. We modeled mortality impacts of current patterns of weather variability by region or city and age group, and applied these relationships to climate and population projections to estimate temperature-related health burdens in UK regions and large Australian cities during the 2020s, 2050s, and 2080s, under three emissions scenarios. Furthermore, we provided a systematic comparison of health burden estimates between the two countries and between age groups.

## Methods

Estimation of mortality associated with temperature consisted of three phases: *a*) time-series regression analyses of daily counts of all-cause mortality and weather factors for UK regions and Australian cities; *b*) assessment of health impacts attributable to heat and cold under current and future climatic conditions; *c*) modeling the contribution of demographic changes on future mortality burdens.

*Statistical time-series analyses*. All deaths in England and Wales from 1993 through 2006 and in the five largest Australian cities with populations > 1 million (Sydney, Melbourne, Brisbane, Adelaide, and Perth) from 1990 through 2006 were obtained from the UK Office for National Statistics (ONS) and the Australian Bureau of Statistics (ABS) respectively. Individual deaths were aggregated to create time series of the daily number of deaths in the study periods. Because the weather can contribute to mortality from many causes, we created series for all-cause mortality (including external causes). This was done by age group (0–64, 65–74, 75–84, ≥ 85 years), for the 10 government regions of England and Wales and the 5 largest Australian cities.

Ambient measurements of temperature and relative humidity were downloaded from the British Atmospheric Data Centre (2014) and the Australian BoM (2014). We created daily mean temperature and relative humidity series that were representative of each region or city, and included only stations with data capture of at least 75% of days during the study periods. Where such data sets from more than one station were available, we calculated daily mean values from these data using weights equal to the populations residing closest to each station. We followed the averaging process and handling of missing values described by Armstrong et al. (2011).

Time-series regression analyses were carried out using Stata (version 12; StataCorp, College Station, TX, USA) to estimate the short-term relationships between daily average temperatures and daily mortality. Poisson variation with scale overdispersion was assumed in all statistical models. Heat and cold effects were estimated separately. For heat, models were restricted to the summer months (i.e., June–September in the United Kingdom and December–March in Australia). Possible long-term trends in mortality were modeled using linear and quadratic terms for time. Intra-annual seasonal patterns in the mortality series were controlled more flexibly using natural cubic splines (NCS) of time with 4 degrees of freedom (df) per summer. NCS were also used to adjust for the estimated effects of daily relative humidity, and day-of-week effects were modeled using six indicator terms. For cold-related mortality assessment, based on all-year models to capture impacts occurring in nonwinter periods also, confounder control was the same as for the heat-related mortality models, but with additional seasonal control (8 df/year) and the inclusion of daily influenza deaths obtained from the UK ONS (comparable influenza data were not available for Australia). Although adjustment for air pollution was not carried out, previous work showed that control for daily particulate matter ≤ 10 μm (PM_10_) and ozone (O_3_) in the London region only changed the estimated relative risk (RR) for heat by a very small amount, whereas the estimated RR for cold remained unchanged (Hajat et al. 2014). Control for daily PM_10_, nitrogen dioxide (NO_2_), or O_3_ in Brisbane did not change the association between temperature and mortality in a previous study (Huang et al. 2012).

Relationships between daily mean temperature and daily mortality, assessed graphically using NCSs of temperature, indicated thresholds in both heat and cold models at which risk increased (data not shown). Therefore, for quantification, we used the 93rd percentile (average of lags 0–1) and the 60th percentile (average of lags 0–27) of the daily mean temperature distribution within each UK region or Australian city as the heat and cold thresholds respectively, based on evidence from earlier studies (Armstrong et al. 2011; Hajat et al. 2014). As a sensitivity test, heat and cold thresholds equivalent to the 90th percentile (average of lags 0–1) and the 65th percentile (average of lags 0–27) of the daily mean temperature distribution within each UK region or Australian city were also used to capture any mortality effects occurring at more moderate temperatures. For the daily mortality and temperature distributions, and thresholds for heat and cold effects in UK regions and Australian cities, see Supplemental Material, Figures S1 and S2. Because heat-related deaths occur soon after exposure (Basu and Samet 2002), they were modeled using the average of same day and previous day temperatures. Cold impacts can be delayed by weeks (Bhaskaran et al. 2010) and therefore were modeled using temperatures averaged over 28 days.

In all models, heat or cold effects are presented as the estimated RR of death for every 1°C increase or decrease in temperature above or below the threshold. RRs are estimated separately by UK region or Australian city, for all ages and separate age groups, as well as a mean RR for each country using a DerSimonian and Laird procedure for a random effects meta-analysis (DerSimonian and Laird 1986).

*Health impact assessment*. Projected monthly mean temperatures for the periods 2020–2029 (2020s), 2050–2059 (2050s), and 2080–2089 (2080s) were obtained from the UK Climate Impacts Programme (UKCIP) Climate Projections (UKCP09 2009) and the Australian Climate Change Scenarios Generator (OzClim 2011). These databases provide output from the Met Office Hadley Centre Climate Model (HadCM3) (Gordon et al. 2000) for the Special Report on Emissions Scenarios (SRES) (Nakicenovic et al. 2000) at a horizontal resolution of approximately 25 km. In this study, three SRES scenarios were used: low (B1), medium (A1B), and high (A1FI) emissions.

Series of daily mean temperatures were calculated separately for each emissions scenario by taking the mean of the monthly temperature for each grid square that fell within a particular regional boundary in England and Wales or by taking the most central grid square for each Australian city, and then estimating the difference from the corresponding average monthly temperature in the same region or city over the baseline period (1993–2006), and applying this difference to the daily mean temperatures over the same period. This produced three daily mean temperature time series (one for each emission scenario) per England and Wales region or Australian city.

Temperature-related mortality was initially calculated for the baseline period (1993–2006) and then for three future decades, 2020s, 2050s, and 2080s, for 10 UK regions and 5 Australian cities using the relationship:


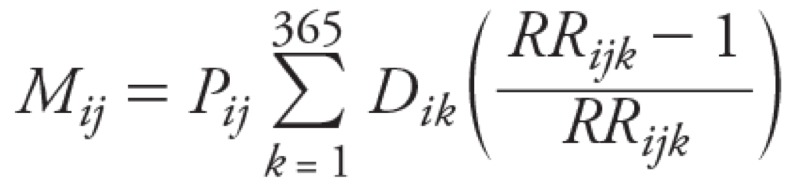
[1]


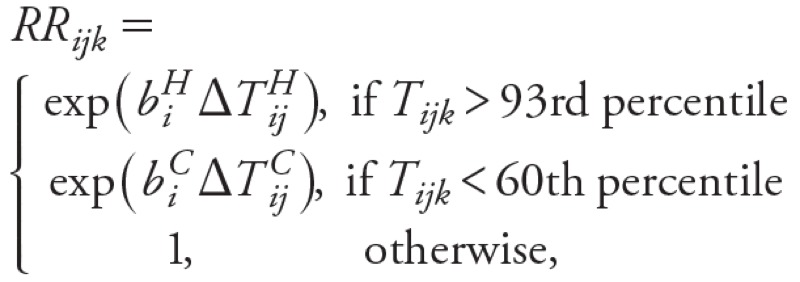
[2]

where *M_ij_* represents the estimated temperature-related deaths per year in region *i* during decade *j*, *P_ij_* the population in region *i* during period *j*, *D_ik_* is the daily mortality rate for all-cause deaths in region *i* on day *k*, *RR_ijk_* is the calculated relative risk for heat or cold effects in region *i* on day *k* of decade *j*, and *b__i__^H^* and *b__i__^C^* are the slopes of the temperature–mortality relationship for heat (*H*) and cold (*C*) respectively in region *i* reflecting the change in all-cause mortality per 1°C change in daily mean temperature above or below the regional threshold for heat or cold derived from the statistical time-series analysis over the baseline period. Finally, Δ*T^^H^^__ijk__* and Δ*T^^C^^__ijk__* are the actual or projected excursions of daily mean temperatures above or below the regional threshold for heat or cold. Mortality burdens were estimated for all ages and for four age groups (0–64, 65–74, 75–84, ≥ 85 years) separately.

The temperature–mortality relationships (i.e., slopes and threshold temperatures) and daily mortality rates were held constant over time, because our main focus was to estimate the effect of changes in temperature on mortality under the three selected emissions scenarios.

*Demographic changes*. Regional population data based on the 2001 census for the United Kingdom and Australia were initially used in the health impact assessment. We subsequently repeated the calculations for future decades using regional population projections. Population data were extracted from the 2010-based principal projections for the United Kingdom (ONS 2011) and from the 2006-based mid-range (Series B) projections for Australia (ABS 2008b), and aggregated into the four age groups for the three decadal periods. The population projections are based on assumptions about future levels of fertility, mortality, internal and overseas migration used by the UK ONS (2011) and the ABS (2008b), which are broadly similar, though not identical, between the countries.

## Results

**Figure 1:**
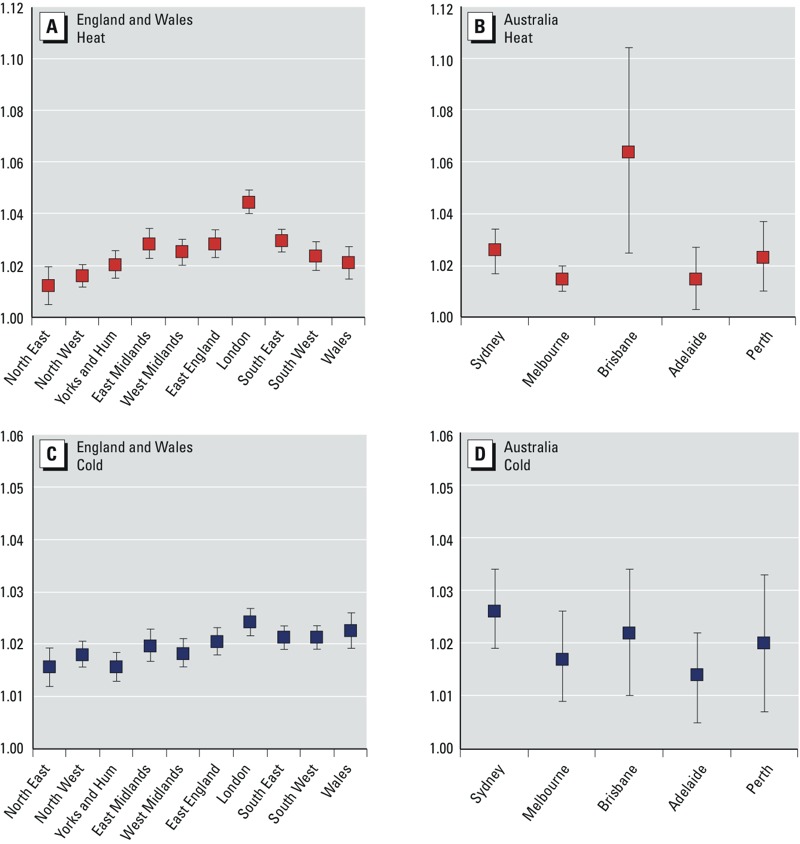
Estimated RRs and 95% CIs for all-cause mortality due to heat (*A,B*; temperature threshold: 93rd percentile, lags 0–1) and cold (*C,D*; temperature threshold: 60th percentile, lags 0–27) for all ages in the England and Wales region (left) and cities of Australia (right). Yorks and Hum, Yorkshire and Humber. The RRs were derived from time-series regression analyses assuming Poisson variation with scale overdispersion. Heat and cold effects were estimated separately.

*Temperature–mortality relationships*. The estimated RR of all-cause mortality increased significantly (*p* < 0.05) when daily mean temperatures exceeded the 93rd percentile thresholds in UK regions and Australian cities (Figure 1). The heat-related RRs showed substantial regional heterogeneity, with people living in London generally having higher RRs than those in other regions of England and Wales (Figure 1). In Australia, there was also substantial heterogeneity among RRs of mortality associated with hot weather in different cities, with Brisbane’s population having higher RR. However, confidence intervals (CIs), particularly for Brisbane but also for other Australian cities (Adelaide and Perth), are very broad because their resident populations are smaller than those of Sydney, Melbourne, London, and other UK regions.

We also estimated statistically significant positive associations between all-cause mortality and daily mean temperatures below the 60th percentile threshold in UK regions and Australian cities (Figure 1). In the United Kingdom, heterogeneity for estimated cold effects was less pronounced than heterogeneity for heat effects, with the population of London having generally higher RRs for cold effects compared with other regions of England and Wales. In Australia, RR CIs were again larger.

**Figure 2:**
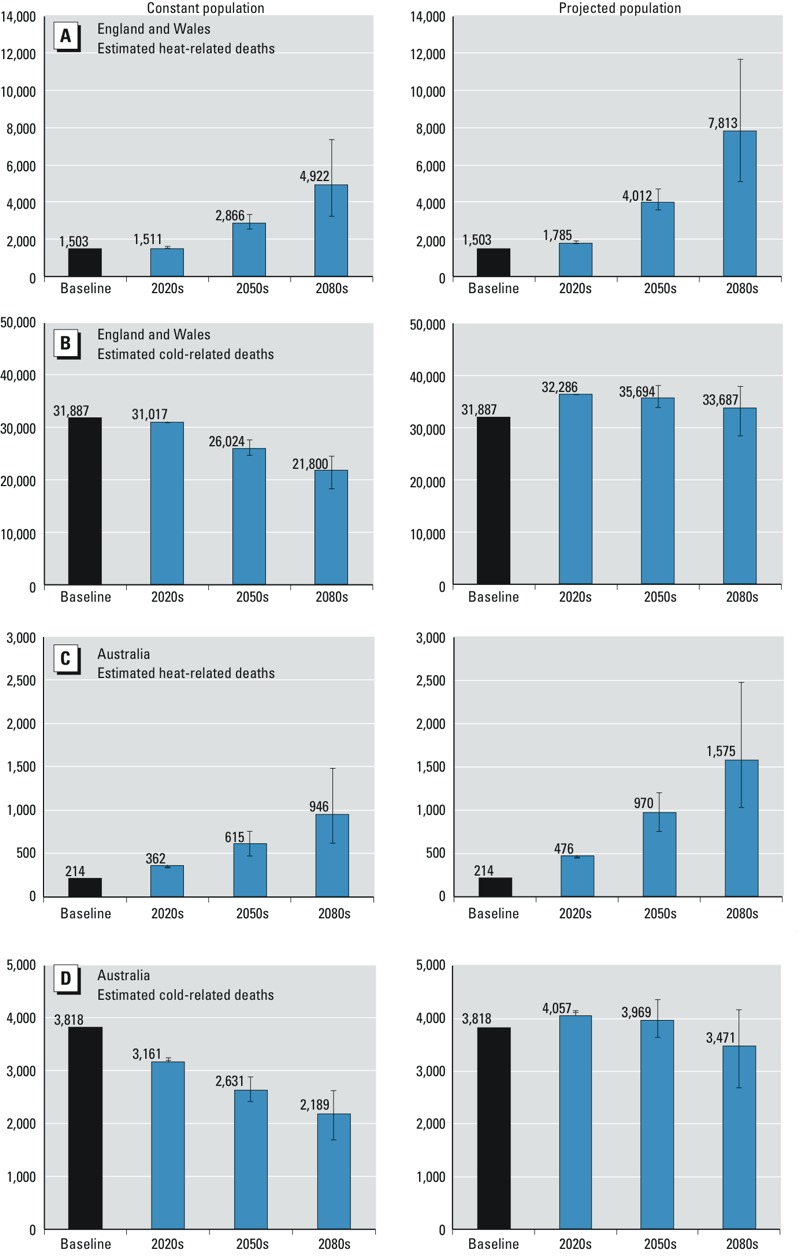
Estimates of heat-related and cold-related deaths in England and Wales regions (*A*,*B*) and five Australian cities (*C*,*D*) per year for all ages for 2001 populations (left) and projected populations (right). The mortality estimates are based on estimated RRs for heat or cold effects in each region, the daily mortality rate for all-cause deaths and population size in each region, and regional daily mean temperatures. Blue bars show estimates based on SRES A1B; B1 and A1FI estimates are shown as error bars. Temperature thresholds: 93rd percentile for heat and 60th percentile for cold. Baseline period: 1993–2006.

For England and Wales, the estimated national-level percentage change in mortality associated with exposure to heat was 2.5% (95% CI: 1.9, 3.1) per 1°C rise in temperature above the heat threshold (93rd percentile of daily mean temperature, average of lags 0–1), whereas for cold it was 2.0% (95% CI: 1.8, 2.2) per 1°C drop in temperature below the cold threshold (60th percentile of daily mean temperature, average of lags 0–27). In the Australian cities, the estimated overall percentage change in mortality associated with exposure to heat was 2.1% (95% CI: 1.3, 2.9), whereas for cold it was 2.0% (95% CI: 1.5, 2.4) for the same percentile thresholds as for England and Wales. Although overall heat and cold-related RR estimates based on observed data for 1993–2006 were broadly similar within each country and between countries, there were more days below the 60th percentile cold threshold than above the 93rd percentile heat threshold, leading to larger numbers of deaths attributable to cold weather (Figure 2). The estimated overall mortality burdens for 1993–2006 in the UK regions were also much larger than those estimated for Australia because of the much larger population size in England and Wales compared with the total for the five Australian cities included in this study (approximately 52 and 12 million in 2001, respectively).

Mortality RR estimates by age group based on observed data for 1993–2006 generally showed significant heat- and cold-related risks in each of the four selected groups (0–64, 65–74, 75–84, ≥ 85 years), focusing on the elderly who are generally more vulnerable to heat and cold temperatures (Hajat et al. 2007) (see Supplemental Material, Table S1). Both heat- and cold-related risks in the United Kingdom and Australia increased with successive age groups, with the greatest risks by far in the group of ≥ 85 years.

*Health impacts*. The estimated mortality burdens associated with hot and cold weather per year in England and Wales regions and Australian cities based on the 93rd and 60th percentile temperature thresholds are presented in Figure 2 for the baseline period (1993–2006) and three future decades (2020s, 2050s, and 2080s). Results are shown for the 2001 populations and projected future populations. For estimated RRs and mortality burdens based on the 90th and 65th percentile temperature thresholds for the same periods, see Supplemental Material, Figures S3 and S4.

For future temperature projections and constant regional populations (2001 census) in England and Wales, the annual mean heat-related mortality was estimated to increase overall by approximately 90% between the 2020s and 2050s (125% with population projections), and by approximately 72% between the 2050s and 2080s (95% with population projections) under the medium emissions scenario (A1B). Cold-related mortality is projected to decrease by around 16% over the 2020s–2050s (2% with population projections) and by another 16% over the 2050s–2080s (6% with population projections) under the same emissions scenario.

In the five Australian cities, for constant population at 2001 levels, the annual mean heat-related mortality is projected to increase overall by approximately 70% between the 2020s and 2050s (104% with population projections), and by approximately 54% between the 2050s and 2080s (62% with population projections) under the medium emissions scenario (A1B). Cold-related mortality is projected to decrease by around 17% over 2020s–2050s (2% with population projections) and by another 17% over 2050s–2080s (13% with population projections) under the same emissions scenario.

Overall, the results suggest that the cold-related mortality burden will remain higher than the heat-related burden in all studied periods in UK regions and Australian cities. However, the ratio between cold- and heat-related deaths will decrease from approximately 20 to 4 between the 2020s and 2080s in the United Kingdom, whereas in Australian cities this decrease will be from approximately 9 to 2 over the same period, under the medium emissions scenario (A1B).

Estimated annual temperature-related deaths by age groups normalized per 100,000 people are presented in Supplemental Material, Figure S5. All results provide strong evidence that the burdens of heat and cold are much higher in age groups 75–84 years and in particular ≥ 85 years in both the United Kingdom and Australia. Heat-related mortality is projected to increase steeply (e.g., by approximately 223% and 163% between the 2020s and 2080s for those ≥ 85 years of age in the United Kingdom and Australia, respectively, for the A1B emission scenario and constant populations) in these two age groups; cold-related mortality is projected to decrease at a substantially lower rate (e.g., by approximately 29% and 30% between the 2020s and 2080s for those ≥ 85 years of age in the United Kingdom and Australia, respectively, for the A1B emission scenario and constant populations) during the same period.

Estimated rates of temperature-related mortality per 100,000 people based on data for 1993–2006 and projections for future decades varied across UK regions and Australian cities (Table 1). In the United Kingdom, London, East and West Midlands, the South East, and East England had the highest rates of heat-related deaths, whereas in Australia the highest rate was in Brisbane. In the United Kingdom, the highest cold-related mortality rates were in Wales, South West, South East, and East England. In Australia, the highest cold-related mortality rate was in Sydney. The regional variations of heat- and cold-related mortality broadly persist in future decades in the two countries. Temperature-related mortality rates are generally lower in Australian cities than in UK regions, with Melbourne having currently the lowest rates for both heat- and cold-related mortality in Australia.

**Table 1:** Heat- and cold-related deaths (n) in England and Wales regions and Australian cities per year per 100,000 population.

Region	Threshold^*a*^	Baseline^*b*^	2020s			2050s			2080s		
			A1B	B1	A1FI	A1B	B1	A1FI	A1B	B1	A1FI
United Kingdom
Heat-related deaths
North East	16.6	1.4	1.0	1.1	1.0	2.0	1.8	2.4	3.5	2.3	5.4
North West	17.3	2.0	1.6	1.7	1.6	2.9	2.6	3.4	5.0	3.3	7.7
Yorkshire and Humber	17.5	2.3	1.7	1.8	1.7	3.1	2.8	3.7	5.4	3.5	8.1
East Midlands	17.8	3.2	3.7	3.9	3.6	6.5	5.9	7.5	10.6	7.2	15.4
West Midlands	17.7	2.9	3.0	3.1	2.9	5.6	4.9	6.5	9.6	6.2	14.3
East England	18.5	2.9	3.1	3.3	3.1	5.9	5.3	6.9	10.0	6.6	14.8
London	19.6	4.7	4.3	4.6	4.3	7.8	7.1	9.1	13.1	8.7	19.3
South East	18.3	3.1	3.8	4.1	3.8	7.5	6.7	8.8	13.0	8.5	19.3
South West	17.6	2.3	2.8	2.9	2.7	5.9	5.0	6.9	10.7	6.6	16.6
Wales	17.2	2.3	1.9	2.1	1.9	4.0	3.4	4.7	7.3	4.4	11.6
All regions		2.9	2.9	3.1	2.9	5.5	4.9	6.4	9.4	6.1	14.1
Cold-related deaths
North East	10.9	50.1	54.8	54.7	54.9	46.4	48.9	44.1	39.3	43.6	33.4
North West	11.9	60.5	61.9	61.8	62.0	52.5	55.5	50.0	44.7	49.5	38.1
Yorkshire and Humber	11.6	51.3	53.5	53.4	53.4	45.4	48.0	43.2	38.5	42.8	32.9
East Midlands	11.8	62.8	57.9	57.7	57.6	48.4	51.6	45.8	40.4	45.5	34.0
West Midlands	11.7	57.7	54.6	54.5	54.5	45.7	48.7	43.3	38.1	42.9	32.0
East England	12.3	63.2	59.0	58.9	58.7	49.6	52.7	46.9	41.5	46.6	35.0
London	13.3	60.5	61.0	60.8	60.7	51.5	54.7	49.0	43.6	48.6	37.1
South East	12.4	64.1	59.8	59.7	59.5	49.8	53.2	47.0	41.4	46.7	34.6
South West	12.2	65.7	60.1	60.1	60.1	49.4	53.0	46.6	40.4	46.0	33.2
Wales	11.9	71.7	71.4	71.3	71.4	59.4	63.4	56.2	49.3	55.6	41.1
All regions		61.0	59.3	59.2	59.2	49.8	52.9	47.2	41.7	46.7	35.1
Australia
Heat-related deaths
Sydney	23.8	1.7	3.3	3.0	3.1	6.2	4.6	7.9	10.1	6.2	16.3
Melbourne	21.5	1.5	2.4	2.3	2.4	3.6	3.0	4.2	5.0	3.6	7.1
Brisbane	26.1	2.9	5.1	4.7	4.9	8.9	6.8	11.1	14.0	8.9	22.5
Adelaide	24.7	1.9	2.2	2.1	2.2	3.0	2.6	3.4	3.8	3.0	5.1
Perth	25.8	1.6	2.4	2.2	2.3	4.0	3.1	4.9	6.1	4.0	9.3
All cities		1.8	3.1	2.9	3.0	5.2	4.1	6.5	8.1	5.3	12.6
Cold-related deaths
Sydney	18.5	41.8	34.3	35.2	34.8	28.8	31.5	26.6	24.2	28.7	19.1
Melbourne	15.2	23.3	17.6	18.1	17.9	14.3	16.0	13.0	11.6	14.3	8.6
Brisbane	22.2	32.2	29.3	30.0	29.7	24.5	26.8	22.5	20.4	24.4	15.8
Adelaide	17.3	29.8	30.0	30.6	30.3	25.6	27.8	23.8	21.9	25.6	17.7
Perth	19.7	30.8	22.9	23.5	23.2	18.7	20.8	17.0	15.1	18.7	11.0
All cities		32.5	26.9	27.6	27.3	22.4	24.6	20.6	18.6	22.4	14.4
Regional estimates of temperature-related deaths per year based on estimated RR*s* for heat or cold effects, the daily mortality rate for all-cause deaths and population size in each region, and projected daily mean temperatures for three SRES emission scenarios (A1B, B1, and A1FI). ^***a***^Temperature thresholds (^o^C) correspond to the 93rd percentile for heat and 60th percentile for cold effects. ^***b***^Baseline period refers to 1993–2006.											

The sensitivity analysis (see Supplemental Material, Figures S3, S4, S6) indicated that the two different sets of temperature thresholds (93rd/60th and 90th/65th percentiles for heat/cold) generally result in comparable estimated mortality burdens in the United Kingdom and Australia for all assessment periods, although there were some noticeable differences (e.g., cold-related deaths in the United Kingdom in the 2080s). However, these differences do not affect the overall interpretation of the results: The same regions and population groups are at highest mortality risk.

## Discussion

*Summary of findings*. This study provides quantitative estimates of current and future temperature-related mortality burdens in two countries, the United Kingdom and Australia, which have broadly similar socioeconomic characteristics but different climates, urban density, and infrastructure. In the absence of any planned, spontaneous behavioral or physiological adaptation of the population to climate change, heat-related mortality is projected to rise steeply (e.g., by approximately 90% and 70% between the 2020s and 2050s in the United Kingdom and Australia, respectively, for constant populations) by mid-century and beyond in both countries because of rising mean temperatures as well as population growth and aging. Over the same period, cold-related mortality estimates show a relatively smaller decline (e.g., by approximately 16% and 17% between the 2020s and 2050s in the United Kingdom and Australia, respectively, for constant populations) that will be largely offset by demographic changes, as suggested by the differences in mortality estimates based on constant versus projected populations (Figure 2). Nevertheless, the absolute number of cold-related deaths is projected to remain higher than that of heat-related deaths in UK regions and Australian cities over the assessment period.

Our results indicate that heat-related mortality RRs in most large Australian cities (Melbourne, Adelaide, Perth, and Sydney) are significantly lower than in London, whereas the estimated RR for Brisbane is higher (but with overlapping CIs) (Figure 1). The contrast in heat-related mortality RRs between London and Melbourne may be due to physiological acclimatization of the population to heat to some extent, but may also be related to spontaneous behavioral (e.g., wearing lighter clothes) and planned adaptation (e.g., use of cooling devices, urban planning, building design, vegetation cover) to higher temperatures (Hajat et al. 2010). These adaptation aspects are potentially transferable to other cities or countries.

The total population of England and Wales is projected to increase from around 52 million in 2001 to around 81 million by the mid-2080s, and in the five Australian cities of this study the population is expected to grow from nearly 12 million in 2001 to nearly 18 million by the mid-2080s. A common characteristic in all UK regions and Australian cities included in this study is the large projected expansion of the ≥ 85 years age group (from around 1.9% of the total population in 2001 to around 8.9% by the mid-2080s in England and Wales, and from around 1.3% of the total population in the five Australian cities to around 6.3% over the same period). The contribution of population growth and aging to future temperature-related mortality is projected to be substantial in UK regions (40% for heat and 37% for cold in 2050s, and 59% for heat and 55% for cold in 2080s) and Australian cities (58% for heat and 51% for cold in 2050s, and 66% for heat and 59% for cold in 2080s) in this century. This estimated demographic effect on mortality is proportionally larger for heat- than for cold-related mortality, and is larger in Australian cities than in UK regions. Better general health and well-being of the elderly, particularly of those living in large cities, as well as planned adaptation measures (e.g., temperature-health warning systems, targeted public health messages, and improved home insulation) may help to alleviate these large mortality burdens (Marmot Review Team 2011).

Our UK estimates of future heat-related mortality burdens broadly agree with previous assessments. Hames and Vardoulakis (2012) estimated an increase of 74% and 57% in annual heat-related mortality in the United Kingdom over the 2020s–2050s and 2050s–2080s, and a decline of 24% and 26% of cold-related mortality over the same periods, based on current population levels. A study by Hajat et al. (2014), based on modeled daily temperature projections for the whole of the UK, estimated increases of 115% and 78% in annual heat-related deaths over the 2020s–2050s and 2050s–2080s, and corresponding decreases of 6% and 10% in cold-related deaths when future population projections were taken into account. However, these two studies were based on different methods for projecting temperatures in UK regions; therefore the results presented here are quantitatively different. Because daily time series of temperature projections were not available for Australia, for consistency we used monthly projections for UK regions and Australian cities. The present study therefore does not take into account any changes in variability in daily mean temperatures from the baseline period, which is particularly important for heat-related health burdens. A study by Armstrong et al. (2011) showed close agreement with the baseline heat-related mortality burden presented in the present study for England and Wales. An earlier study from Australia reported an increase in heat-related mortality of around 15% by 2070 (Bambrick et al. 2008).

*Adaptation to higher temperatures*. In this study we have estimated recent and future temperature-related mortality assuming a stationary relationship between daily deaths and mean temperature in different regions or cities in the United Kingdom and Australia. This approach, assuming that future temperature–mortality relationships are identical to past ones, could overestimate the health burden of heat as people will progressively acclimatize and adapt, to some extent, to rising temperatures (Gosling et al. 2009a).

In earlier studies, the analog city approach has been used to model the effect of adaptation in cities within the same country (Kalkstein and Greene 1997; Knowlton et al. 2007). Although Melbourne appears to be an appropriate analog city for London on the basis of projected temperatures and socioeconomic status [gross domestic product per capita (purchasing power parity): US$42,700 in Greater London compared with US$37,100 in Greater Melbourne in 2005], population density differs substantially between the two cities (5,200/km^2^ in Greater London compared with only 430/km^2^ in Greater Melbourne in 2012). Furthermore, in Greater Melbourne around 23% of all dwellings were single-person households, around 15% were flats (units and apartments), and around two-thirds of all households had at least one air-conditioning unit used for cooling in 2011. By comparison, in Greater London around 32% of all dwellings were single-person households, around 50% were flats (purpose built or converted), and the vast majority of all households did not use mechanical cooling devices.

In Victoria, and similarly across the rest of Australia, the proportion of households with an air conditioner in use almost doubled from 1994 to 2008 (ABS 2008a). Air conditioning is likely to become more widely used in London and other UK cities. Technological innovation and environmental considerations may also lead to a wider implementation of passive cooling techniques, including building shading, thermal insulation, green roofs and walls, and novel construction materials and paints (Vardoulakis and Heaviside 2012). However, because existing urban structures cannot be greatly modified to adapt to climate change, populations would be unable to fully adapt to rising temperatures (Kalkstein and Greene 1997). Increasing population density and social isolation would slow down adaptation, whereas improvements in health care and housing conditions would have the opposite effect.

Future severe weather protection plans and warning systems are expected to mitigate temperature-related mortality in the United Kingdom and Australia to some extent, although there is still limited quantitative evidence on their efficacy. Heat wave plans are in operation in England and Wales (e.g., Public Health England 2014), and in some Australian states (e.g., State of Victoria Department of Health 2011). Longer-term adaptation, including sustainable urban planning, climate-resilient infrastructure, effective emergency management and response, community engagement and participation, and public health education and promotion are also expected to reduce vulnerability to severe weather (Hames and Vardoulakis 2012).

*Methodological considerations*. In the present study, we have used regional or city-level daily mortality and temperature data over a relatively long baseline period (14–17 years), projected temperatures for three future decades based on three climatic scenarios covering a range of possible futures, and analyzed four age groups reflecting current and future demographics. The same methods and models have been consistently used as far as possible in our analyses for the United Kingdom and Australia, allowing a meaningful comparison of the results obtained.

We calculated temperature-related mortality based on a uniform monthly increase of daily mean temperatures observed over the baseline period. This did not account for a potential increase in the frequency of heat waves during summer, which has been projected for temperate climatic zones over this century (Meehl and Tebaldi 2004). Hames and Vardoulakis (2012) assessed the potential impact of a higher frequency of heat waves on mortality in the United Kingdom by using a 4-year baseline period that included 2 major heat wave years (2003 and 2006). Their analysis resulted in 18–33% higher heat-related mortality estimates over the 21st century, compared with estimates obtained using a considerably longer baseline period (1993–2006).

We have presented estimates of uncertainty in our results based on the three climatic scenarios used. However, a full uncertainty analysis involving uncertainties in climate model physics and population projections has not been undertaken. Hajat et al. (2014) projected annual temperature-related mortality in the United Kingdom based on nine variants of a climate model for a medium emission scenario. The spread of their model results, reflecting uncertainty in climate model physics, was substantially larger than the uncertainty associated with different emissions scenarios reported here.

An additional consideration relates to the definition of temperature thresholds below or above which effects on daily mortality become apparent. We have used the 93rd and 60th percentiles of daily mean temperatures over the whole year in each region or city as thresholds for heat and cold effects, respectively. We also used a set of more pessimistic temperature thresholds (90th and 65th percentiles of daily mean temperatures for heat and cold effects, respectively) for each city or region as a sensitivity test. Thresholds for heat effects used in other UK studies are consistently above the 90th percentile of daily (maximum or mean) temperatures (lags 0–1), whereas thresholds for cold effects tend not to be as well defined as those for heat (Armstrong et al. 2011; Donaldson et al. 2002; Hajat et al. 2007, 2014; Pattenden et al. 2003). Hames and Vardoulakis (2012) carried out a sensitivity analysis using thresholds corresponding to daily mean temperature percentiles from 5th to 90th, which resulted in a wide range of projected cold-related mortality estimates for the United Kingdom over the 21st century. Also, some of the estimated temperature-related mortality may arise as a result of short-term mortality displacement (harvesting), whereby the deaths of already frail individuals are brought forward by only a few days or weeks because of exposure to hot or cold weather (Hajat et al. 2005). Previous work has indicated that some heat-related deaths may be explained by short-term mortality displacement, but this is less clear in the case of cold-related deaths (Braga et al. 2002).

Finally, we have chosen not to model the additional effect of heat waves (i.e., consecutive days of extremely hot weather) over and above the general effect of temperature on mortality, as included in some other studies (Hajat et al. 2014; Peng et al. 2011), because such events are responsible for only a relatively small fraction of the total current heat burden (Hajat et al. 2006).

## Conclusions

Comparisons between temperature-related mortality estimates from different countries are difficult due to variations in the use of emissions scenarios, climate models, future adaptation and demographic assumptions, and modeling choices in epidemiological time-series analyses. In this study, we have followed the same methods for assessing heat- and cold-related mortality in UK regions and Australian cities over the 21st century under three climate change scenarios.

Both countries experience substantial temperature-related mortality burdens every year, with the number of deaths attributable to cold currently much higher than the number of those due to heat. In all regions and cities included in the analysis, the elderly (particularly those ≥ 85 years old) were most at risk. London appeared more vulnerable to both hot and cold weather compared with most other regions in England and Wales, whereas the population of Melbourne appeared to be better protected from heat. Geographical heterogeneity in these risk estimates may reflect differences in demographic, health, behavioral, urban, and built-environment characteristics. Projected changes in climate are likely to lead to a steep increase in heat-related mortality in the United Kingdom and Australia over this century, but also in a proportionally smaller decrease in cold-related deaths. The absolute number of cold-related deaths is projected to remain higher than that of heat-related deaths in both countries over the assessment period, indicating that interventions to reduce cold-related mortality are needed now and will be useful for decades to come. However, health protection from hot weather will become increasingly necessary. Future temperature-related health burdens will be amplified by aging populations.

This study indicates that percentile temperature thresholds for heat and cold effects (see Supplemental Material, Figure S1) are consistent between two countries with very different temperature regimes but similar socioeconomic characteristics. Differences in RR estimates between cities or regions (within the same country or in different countries) can provide insights into effective adaptation to climate change and health protection against severe weather. There is a clear research need to further characterize the health effects of heat and cold taking population acclimatization and adaptation into account. This will enable evidence-based assessments for climate change adaption planning.

## Supplemental Material

(629 KB) PDF
