# Erratum: “Comparative Assessment of the Effects of Climate Change on Heat- and Cold-Related Mortality in the United Kingdom and Australia”

**DOI:** 10.1289/ehp.122-A320

**Published:** 2014-12-01

In the Advance Publication of “Comparative Assessment of the Effects of Climate Change on Heat- and Cold-Related Mortality in the United Kingdom and Australia” by Vardoulakis et al. [Environ Health Perspect 122:1285–1292 (2014); http://dx.doi.org/10.1289/ehp.1307524], Anthony J. McMichael was inadvertently omitted from the list of authors. His name has been included in the final version.

The authors regret the error.

