# Supplementary material for: Comparative Assessment of the Effects of Climate Change on Heat- and Cold-Related Mortality in the United Kingdom and Australia
Source: Environ Health Perspect. 2014 Sep 15;122(12):1285–92. doi: 10.1289/ehp.1307524 (PMC4256046; doi:10.1289/ehp.1307524)
Supplement: (629 KB) PDF [file ehp.1307524.s001.508.pdf]

Environmental Health Perspectives doi:10.1289/ehp.1307524

Supplemental Materials

Comparative Assessment of the Effects of Climate Change on Heat- and Cold-Related Mortality in the United Kingdom and Australia

Sotiris Vardoulakis, Keith Dear, Shakoor Hajat, Clare Heaviside, Bernd Eggen, and Anthony J. McMichael*

*Deceased

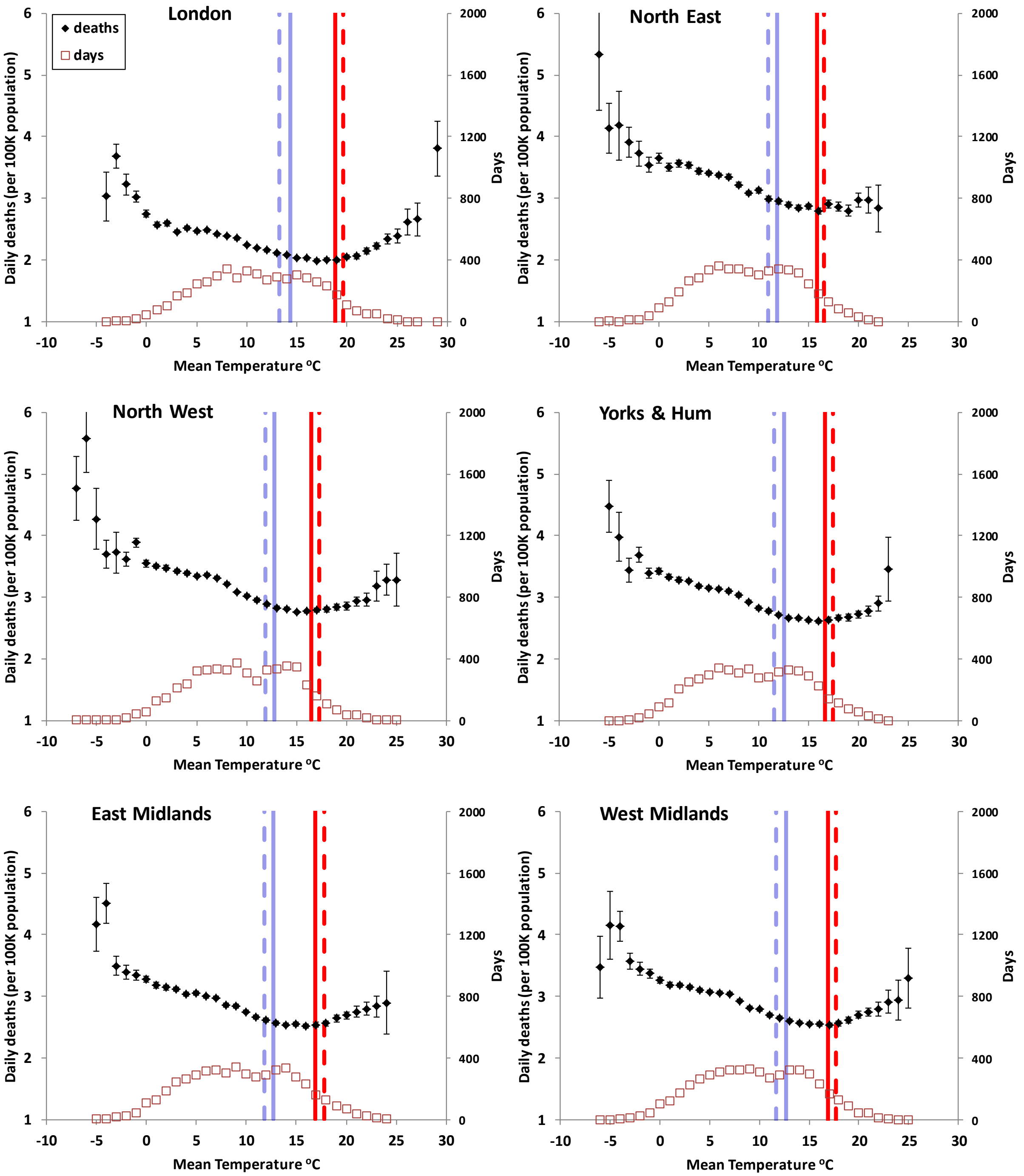
London
deaths
days
North East
North West
Yorks & Hum
East Midlands
West Midlands
Daily deaths (per 100K population)
Days
Mean Temperature °C
-10
-5
0
5
10
15
20
25
30
1
2
3
4
5
6
0
400
800
1200
1600
2000

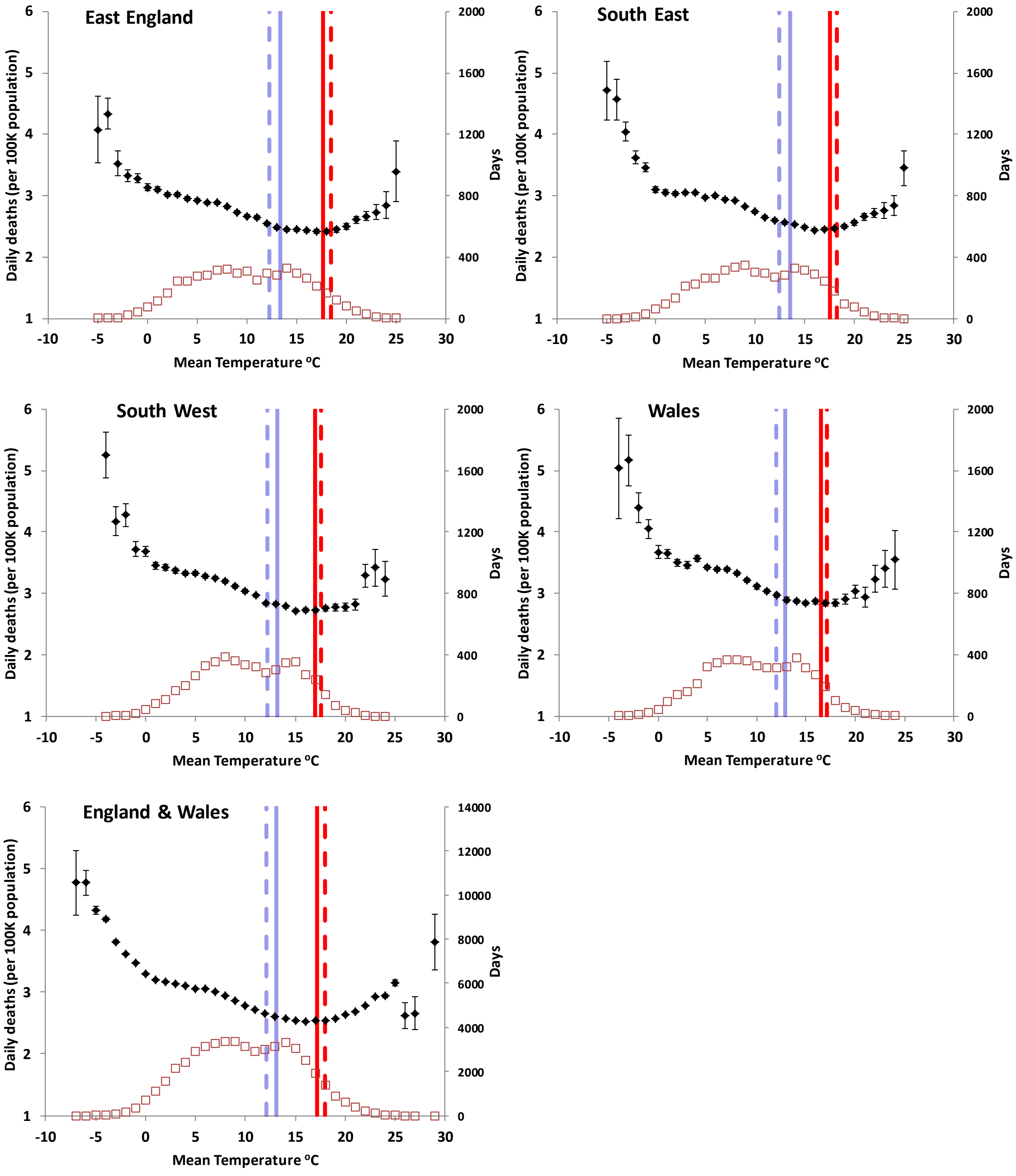

Figure S1. Mean daily deaths (per 100,000 population) and aggregate number of days associated with daily mean temperature in England and Wales regions. Cold and hot temperature thresholds are indicated: (a) 60th percentile (average of lags 0–27) and 93rd percentile (average of lags 0–1) of daily mean temperatures (**dashed lines**); (b) 65th percentile (average of lags 0–27) and 90th percentile (average of lags 0–1) of daily mean temperatures (**continuous lines**).

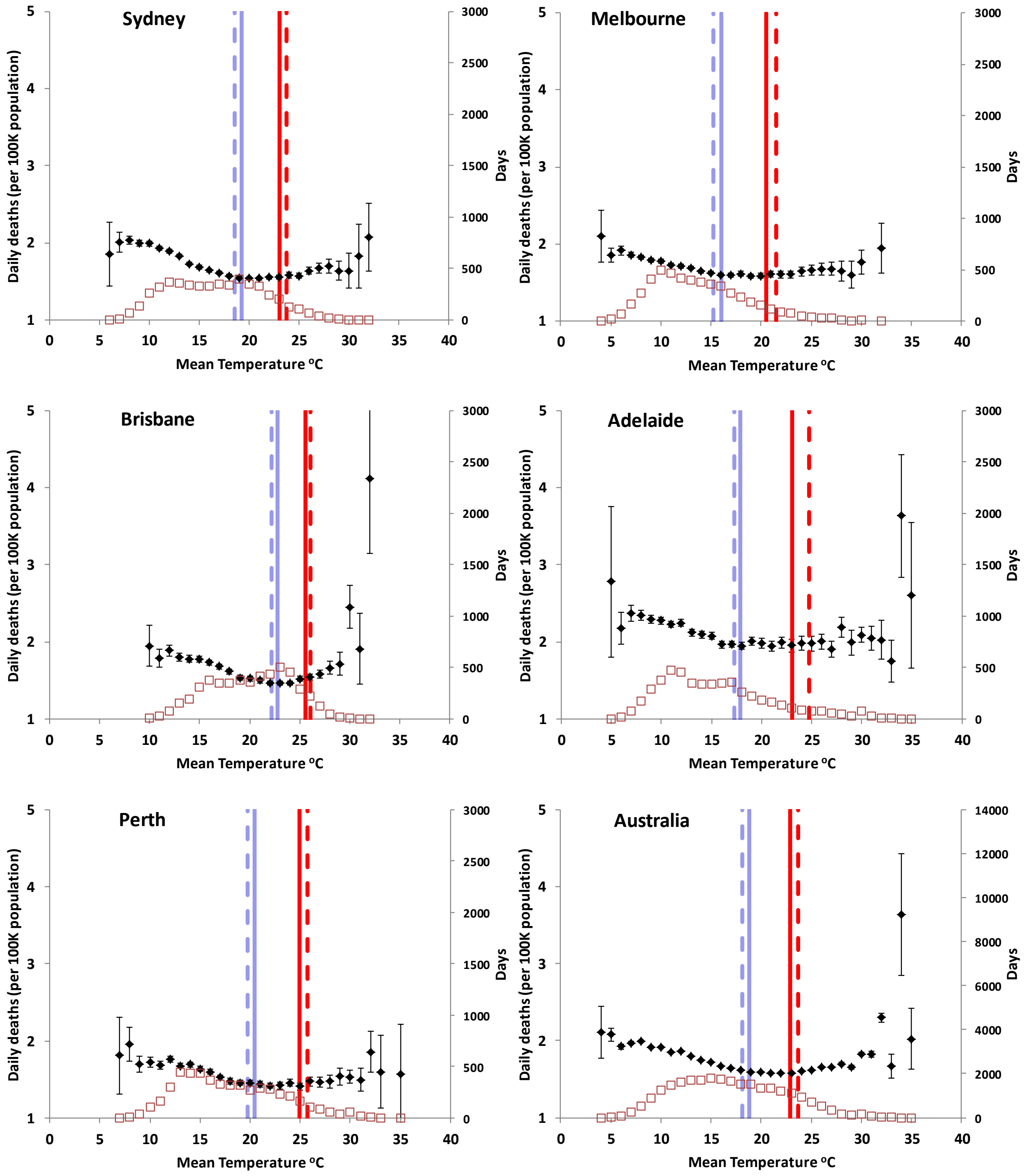

Figure S2. Mean daily deaths (per 100,000 population) and aggregate number of days associated with daily mean temperature in Australian cities. Cold and hot temperature thresholds are indicated: (a) 60th percentile (average of lags 0–27) and 93rd percentile (average of lags 0–1) of daily mean temperatures (**dashed lines**); (b) 65th percentile (average of lags 0–27) and 90th percentile (average of lags 0–1) of daily mean temperatures (**continuous lines**).

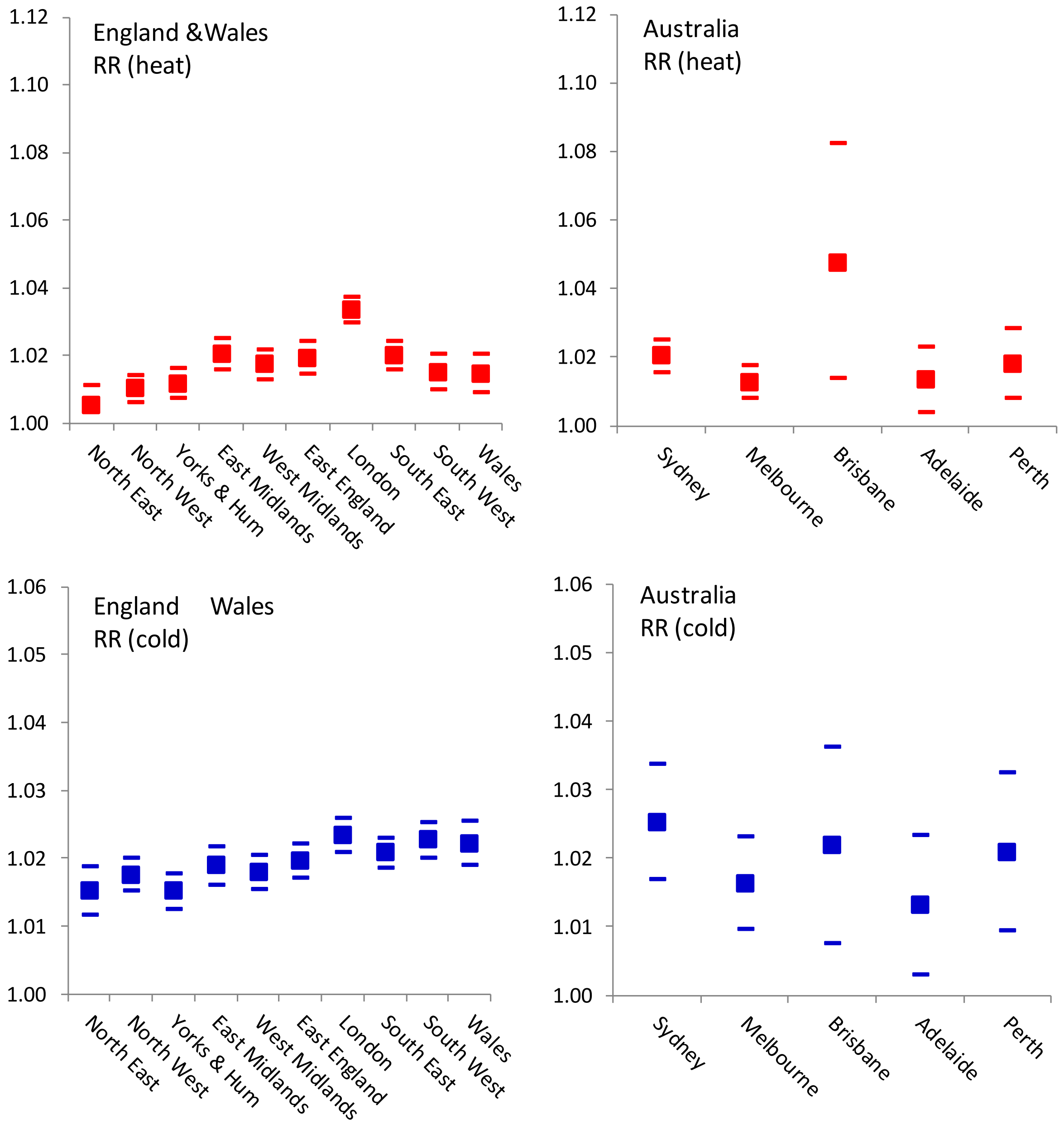

Figure S3. England and Wales region (left) or Australian city (right) estimated relative risks and 95% confidence intervals for all-cause mortality due to heat (top, temperature threshold: 90rd percentile, lags 0–1) and cold (bottom, temperature threshold: 65th percentile, lags 0–27) for all ages. The *RRs* were derived from time-series regression analyses assuming Poisson variation with scale overdispersion. Heat and cold effects were estimated separately.

Table S1. Estimated relative risks and 95% confidence intervals for all-cause mortality due to cold and hot weather for different age groups in England and Wales regions and Australian cities for two sets of temperature thresholds: 60th and 93rd percentiles; and 65th and 90th percentiles.

**Location and age group**	**Cold (< 60th %ile) *RR* (95% CI)**	**Heat (> 93rd %ile) *RR* (95% CI)**	**Cold (< 65th %ile) *RR* (95% CI)**	**Heat (> 90rd %ile) *RR* (95% CI)**
**UK**				
All ages				
North East	1.016 (1.012, 1.019)	1.012 (1.005, 1.019)	1.015 (1.012, 1.019)	1.006 (1.000, 1.011)
North West	1.018 (1.016, 1.021)	1.016 (1.012, 1.020)	1.018 (1.015, 1.020)	1.010 (1.006, 1.014)
Yorks & Hum	1.016 (1.013, 1.019)	1.020 (1.015, 1.026)	1.015 (1.012, 1.018)	1.012 (1.007, 1.017)
East Midlands	1.020 (1.017, 1.023)	1.029 (1.023, 1.034)	1.019 (1.016, 1.022)	1.021 (1.016, 1.025)
West Midlands	1.018 (1.016, 1.021)	1.025 (1.020, 1.030)	1.018 (1.015, 1.021)	1.017 (1.013, 1.022)
East England	1.020 (1.018, 1.023)	1.028 (1.023, 1.034)	1.020 (1.017, 1.022)	1.020 (1.015, 1.024)
London	1.024 (1.022, 1.027)	1.045 (1.040, 1.049)	1.024 (1.021, 1.026)	1.034 (1.030, 1.038)
South East	1.021 (1.019, 1.024)	1.030 (1.025, 1.034)	1.021 (1.019, 1.023)	1.020 (1.016, 1.024)
South West	1.021 (1.019, 1.024)	1.024 (1.018, 1.029)	1.023 (1.020, 1.025)	1.015 (1.010, 1.021)
Wales	1.023 (1.019, 1.026)	1.021 (1.015, 1.027)	1.022 (1.019, 1.026)	1.015 (1.009, 1.020)
0-64				
North East	1.007 (0.999, 1.015)	0.997 (0.981, 1.013)	1.006 (0.998, 1.014)	0.992 (0.981, 1.004)
North West	1.009 (1.004, 1.014)	1.014 (1.005, 1.023)	1.008 (1.003, 1.013)	1.012 (1.005, 1.019)
Yorks & Hum	1.003 (0.997, 1.009)	1.005 (0.992, 1.017)	1.002 (0.996, 1.008)	1.002 (0.993, 1.011)
East Midlands	1.011 (1.005, 1.018)	1.018 (1.005, 1.031)	1.011 (1.004, 1.017)	1.011 (1.002, 1.021)
West Midlands	1.007 (1.002, 1.013)	1.018 (1.007, 1.028)	1.007 (1.001, 1.012)	1.015 (1.007, 1.024)
East England	1.010 (1.004, 1.016)	1.022 (1.009, 1.035)	1.009 (1.003, 1.014)	1.014 (1.004, 1.023)
London	1.012 (1.007, 1.017)	1.028 (1.019, 1.037)	1.011 (1.006, 1.016)	1.022 (1.015, 1.029)
South East	1.011 (1.007, 1.016)	1.015 (1.004, 1.025)	1.009 (1.004, 1.014)	1.009 (1.001, 1.017)
South West	1.015 (1.008, 1.021)	1.008 (0.994, 1.022)	1.013 (1.006, 1.019)	1.006 (0.995, 1.017)
Wales	1.007 (1.000, 1.015)	1.000 (0.985, 1.015)	1.006 (0.999, 1.014)	1.001 (0.990, 1.013)

**Location and age group**	**Cold (< 60th %ile)** ***RR*** **(95% CI)**	**Heat (> 93rd %ile)** ***RR*** **(95% CI)**	**Cold (< 65th %ile)** ***RR*** **(95% CI)**	**Heat (> 90rd %ile)** ***RR*** **(95% CI)**
65-74				
North East	1.015 (1.008, 1.022)	1.021 (1.007, 1.036)	1.015 (1.008, 1.022)	1.012 (1.001, 1.023)
North West	1.013 (1.009, 1.018)	1.006 (0.997, 1.015)	1.013 (1.008, 1.017)	1.004 (0.997, 1.011)
Yorks & Hum	1.019 (1.013, 1.024)	1.024 (1.013, 1.036)	1.017 (1.012, 1.022)	1.011 (1.003, 1.019)
East Midlands	1.012 (1.006, 1.017)	1.015 (1.003, 1.027)	1.011 (1.006, 1.017)	1.009 (1.000, 1.018)
West Midlands	1.016 (1.011, 1.021)	1.024 (1.014, 1.034)	1.014 (1.009, 1.019)	1.014 (1.006, 1.023)
East England	1.014 (1.009, 1.019)	1.016 (1.005, 1.028)	1.014 (1.008, 1.019)	1.013 (1.004, 1.022)
London	1.023 (1.018, 1.028)	1.025 (1.016, 1.034)	1.021 (1.016, 1.026)	1.018 (1.011, 1.025)
South East	1.016 (1.012, 1.020)	1.025 (1.016, 1.035)	1.013 (1.009, 1.017)	1.015 (1.007, 1.022)
South West	1.020 (1.014, 1.025)	1.023 (1.010, 1.036)	1.017 (1.011, 1.022)	1.015 (1.005, 1.025)
Wales	1.009 (1.002, 1.016)	1.015 (1.002, 1.029)	1.009 (1.002, 1.015)	1.009 (0.998, 1.020)
75-84				
North East	1.022 (1.016, 1.028)	1.020 (1.008, 1.033)	1.022 (1.016, 1.028)	1.012 (1.002, 1.021)
North West	1.022 (1.018, 1.026)	1.010 (1.003, 1.018)	1.021 (1.018, 1.025)	1.006 (1.000, 1.012)
Yorks & Hum	1.018 (1.013, 1.022)	1.016 (1.008, 1.025)	1.017 (1.013, 1.021)	1.007 (1.000, 1.014)
East Midlands	1.024 (1.020, 1.029)	1.032 (1.022, 1.041)	1.023 (1.019, 1.028)	1.023 (1.016, 1.030)
West Midlands	1.018 (1.013, 1.022)	1.023 (1.015, 1.031)	1.017 (1.013, 1.021)	1.015 (1.009, 1.022)
East England	1.020 (1.016, 1.024)	1.029 (1.020, 1.038)	1.019 (1.015, 1.023)	1.019 (1.012, 1.026)
London	1.025 (1.021, 1.029)	1.048 (1.041, 1.056)	1.023 (1.019, 1.027)	1.036 (1.030, 1.042)
South East	1.025 (1.022, 1.029)	1.021 (1.014, 1.028)	1.022 (1.019, 1.026)	1.014 (1.008, 1.020)
South West	1.026 (1.022, 1.031)	1.026 (1.016, 1.035)	1.023 (1.019, 1.028)	1.015 (1.007, 1.023)
Wales	1.030 (1.025, 1.036)	1.022 (1.012, 1.033)	1.028 (1.023, 1.033)	1.016 (1.007, 1.025)

Location and age group	Cold (< 60th %ile) *RR* (95% CI)	Heat (> 93rd %ile) *RR* (95% CI)	Cold (< 65th %ile) *RR* (95% CI)	Heat (> 90rd %ile) *RR* (95% CI)
85+				
North East	1.016 (1.009, 1.023)	1.002 (0.987, 1.016)	1.014 (1.007, 1.021)	1.000 (0.990, 1.011)
North West	1.025 (1.021, 1.029)	1.031 (1.023, 1.039)	1.024 (1.020, 1.028)	1.019 (1.013, 1.026)
Yorks & Hum	1.021 (1.016, 1.026)	1.033 (1.023, 1.043)	1.019 (1.015, 1.024)	1.025 (1.017, 1.033)
East Midlands	1.026 (1.021, 1.031)	1.042 (1.032, 1.052)	1.025 (1.020, 1.030)	1.033 (1.025, 1.041)
West Midlands	1.031 (1.027, 1.036)	1.034 (1.025, 1.043)	1.029 (1.025, 1.034)	1.024 (1.016, 1.031)
East England	1.031 (1.026, 1.035)	1.038 (1.029, 1.047)	1.029 (1.025, 1.033)	1.028 (1.020, 1.036)
London	1.035 (1.030, 1.039)	1.064 (1.057, 1.072)	1.034 (1.030, 1.038)	1.050 (1.043, 1.056)
South East	1.029 (1.026, 1.033)	1.047 (1.040, 1.054)	1.028 (1.025, 1.031)	1.034 (1.027, 1.040)
South West	1.031 (1.027, 1.036)	1.029 (1.019, 1.038)	1.030 (1.025, 1.034)	1.020 (1.012, 1.028)
Wales	1.038 (1.032, 1.044)	1.034 (1.022, 1.046)	1.035 (1.029, 1.041)	1.025 (1.015, 1.035)
**Australia**				
All ages				
Sydney	1.026 (1.019, 1.034)	1.026 (1.017, 1.034)	1.025 (1.017, 1.034)	1.021 (1.016, 1.025)
Melbourne	1.017 (1.009, 1.026)	1.015 (1.010, 1.020)	1.016 (1.010, 1.023)	1.013 (1.008, 1.018)
Brisbane	1.022 (1.010, 1.034)	1.064 (1.025, 1.104)	1.022 (1.008, 1.036)	1.048 (1.014, 1.083)
Adelaide	1.014 (1.005, 1.022)	1.015 (1.003, 1.027)	1.013 (1.003, 1.023)	1.014 (1.004, 1.023)
Perth	1.020 (1.007, 1.033)	1.023 (1.010, 1.037)	1.021 (1.009, 1.032)	1.018 (1.008, 1.028)
0-64				
Sydney	1.013 (0.998, 1.029)	1.012 (1.003, 1.022)	1.013 (0.999, 1.027)	1.007 (1.000, 1.014)
Melbourne	1.018 (0.996, 1.041)	1.017 (1.006, 1.029)	1.014 (0.996, 1.033)	1.015 (1.006, 1.025)
Brisbane	1.002 (0.976, 1.028)	1.019 (0.967, 1.073)	0.998 (0.974, 1.022)	1.019 (0.979, 1.061)
Adelaide	1.004 (0.976, 1.032)	1.017 (1.001, 1.034)	1.011 (0.987, 1.035)	1.018 (1.006, 1.030)
Perth	1.000 (0.977, 1.022)	1.026 (1.007, 1.045)	1.007 (0.982, 1.032)	1.013 (1.000, 1.027)
65-74				
Sydney	1.018 (0.999, 1.038)	1.018 (1.004, 1.032)	1.018 (1.001, 1.035)	1.016 (1.004, 1.029)
Melbourne	1.008 (0.987, 1.029)	1.006 (0.995, 1.017)	1.007 (0.990, 1.025)	1.007 (0.997, 1.016)
Brisbane	1.031 (1.009, 1.053)	1.077 (1.018, 1.140)	1.022 (1.000, 1.045)	1.056 (1.015, 1.098)
Adelaide	1.020 (0.997, 1.044)	1.018 (1.000, 1.037)	1.001 (0.974, 1.029)	1.014 (0.999, 1.029)
Perth	1.031 (1.009, 1.052)	1.021 (0.997, 1.045)	1.035 (1.012, 1.059)	1.011 (0.995, 1.027)

Location and age group	Cold (< 60th %ile) *RR* (95% CI)	Heat (> 93rd %ile) *RR* (95% CI)	Cold (< 65th %ile) *RR* (95% CI)	Heat (> 90rd %ile) *RR* (95% CI)
75-84				
Sydney	1.029 (1.016, 1.042)	1.027 (1.012, 1.042)	1.026 (1.014, 1.038)	1.023 (1.013, 1.032)
Melbourne	1.017 (1.007, 1.028)	1.013 (1.001, 1.025)	1.016 (1.007, 1.024)	1.010 (1.001, 1.019)
Brisbane	1.003 (0.967, 1.039)	1.076 (1.033, 1.121)	1.013 (0.977, 1.050)	1.051 (1.011, 1.093)
Adelaide	1.018 (1.002, 1.035)	1.019 (0.996, 1.043)	1.020 (1.005, 1.034)	1.016 (1.000, 1.033)
Perth	1.023 (1.003, 1.045)	1.045 (1.028, 1.062)	1.022 (0.999, 1.045)	1.033 (1.019, 1.048)
85+				
Sydney	1.042 (1.028, 1.057)	1.043 (1.024, 1.061)	1.040 (1.027, 1.053)	1.033 (1.019, 1.047)
Melbourne	1.023 (1.008, 1.039)	1.024 (1.014, 1.035)	1.025 (1.014, 1.037)	1.018 (1.007, 1.029)
Brisbane	1.054 (1.024, 1.086)	1.088 (1.013, 1.168)	1.050 (1.019, 1.082)	1.062 (1.005, 1.122)
Adelaide	1.011 (0.989, 1.032)	1.010 (0.997, 1.023)	1.015 (0.991, 1.041)	1.007 (0.997, 1.016)
Perth	1.024 (1.001, 1.047)	1.011 (0.988, 1.034)	1.023 (1.001, 1.045)	1.010 (0.993, 1.027)

The *RRs* were derived from time-series regression analyses assuming Poisson variation with scale overdispersion. Heat and cold effects were estimated separately.

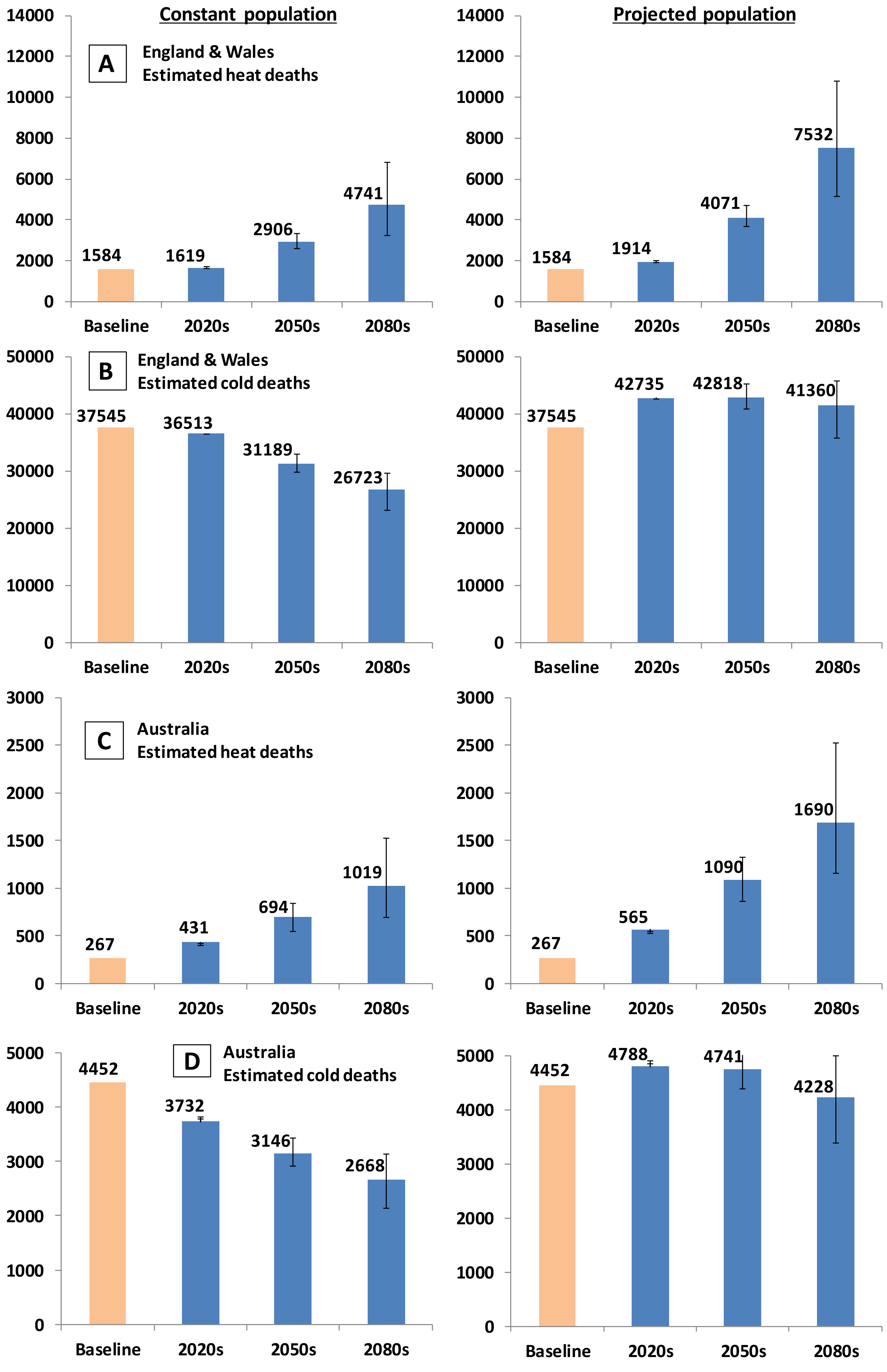

Constant population
Projected population
A
England & Wales
Estimated heat deaths
1584
1619
2906
4741
1914
4071
7532
B
England & Wales
Estimated cold deaths
37545
36513
31189
26723
42735
42818
41360
C
Australia
Estimated heat deaths
267
431
694
1019
565
1090
1690
D
Australia
Estimated cold deaths
4452
3732
3146
2668
4788
4741
4228
Baseline
2020s
2050s
2080s

Figure S4. Heat-related and cold-related deaths in England and Wales regions (A, B) and five Australian cities (C, D) per year for all ages, for 2001 populations (left panel) and projected populations (right panel). The mortality estimates are based on estimated *RRs* for heat or cold effects in each region, the daily mortality rate for all-cause deaths and population size in each region, and regional daily mean temperatures. Coloured bars show estimates based on SRES A1B; B1 and A1FI estimates are shown as vertical lines. Temperature thresholds: 90th percentile for heat and 65th percentile for cold. Baseline period: 1993-2006.

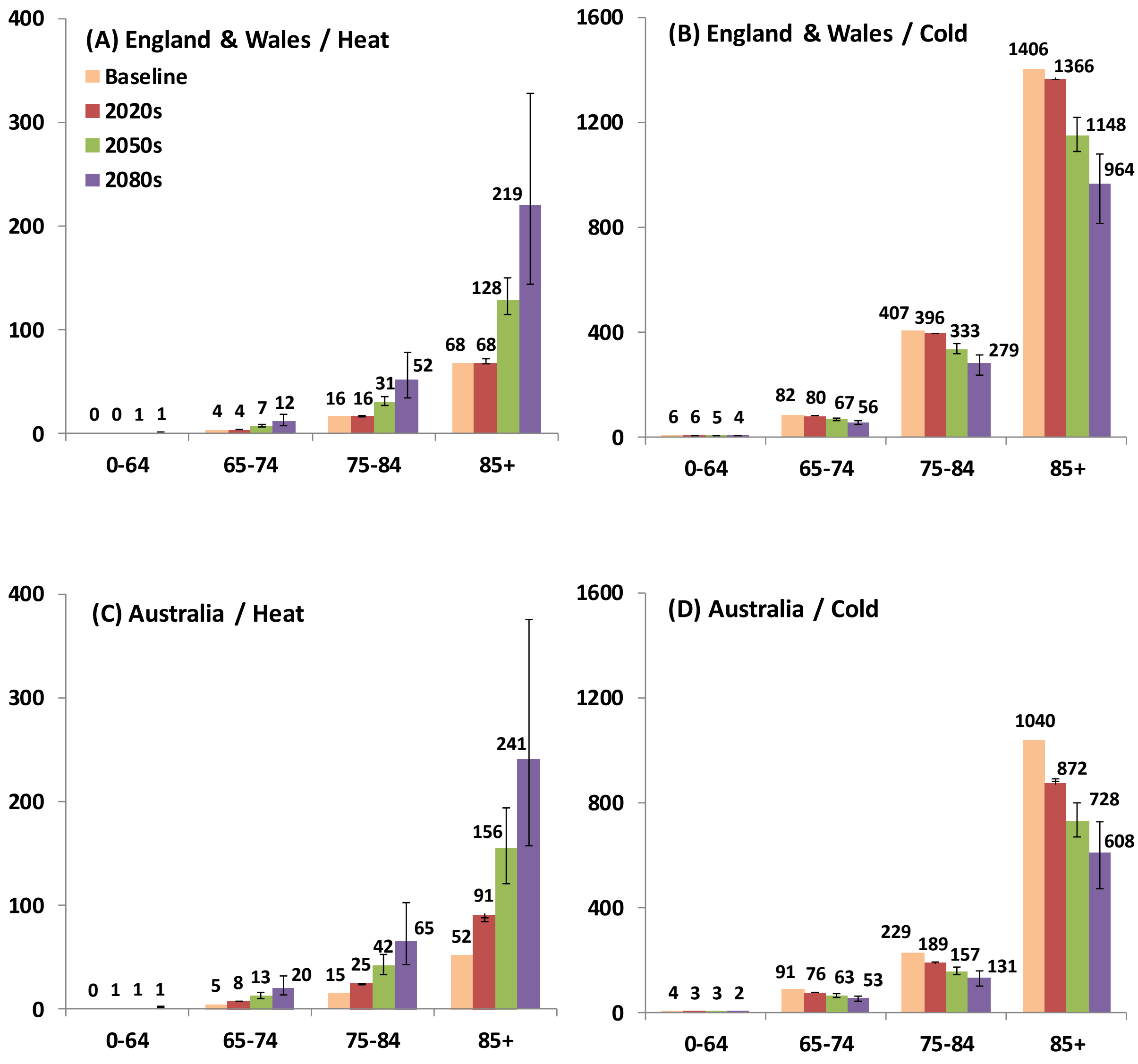

Figure S5. Temperature-related deaths in England and Wales regions (A, B) and Australian cities (C, D) per year, per 100,000 population and by age group for heat (left panel) and cold (right panel). The mortality estimates are based on estimated *RRs* for heat or cold effects for the four age groups in each region, the daily mortality rate for all-cause deaths and population size of each age group in each region, and regional daily mean temperatures. Coloured bars show estimates based on SRES A1B; B1 and A1FI estimates are shown as vertical lines. Temperature thresholds: 93rd percentile for heat and 60th percentile for cold. Baseline period: 1993-2006.

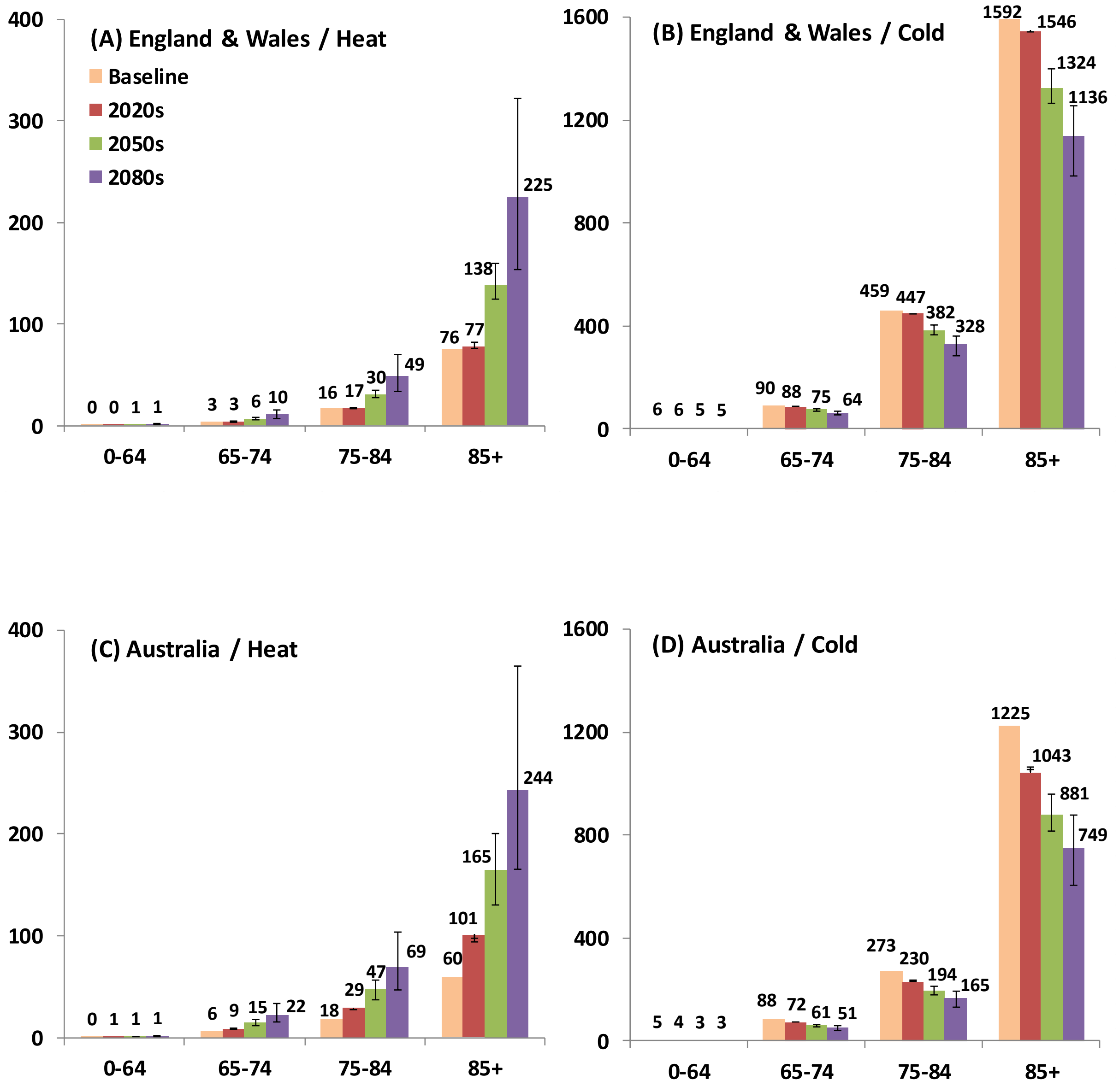

Figure S6. Temperature-related deaths in England and Wales regions (A, B) and Australian cities (C, D) per year, per 100,000 population and by age group for heat (left panel) and cold (right panel). The mortality estimates are based on estimated *RRs* for heat or cold effects for the four age groups in each region, the daily mortality rate for all-cause deaths and population size of each age group in each region, and regional daily mean temperatures. Coloured bars show estimates based on SRES A1B; B1 and A1FI estimates are shown as vertical lines. Temperature thresholds: 90th percentile for heat and 65th percentile for cold. Baseline period: 1993-2006.
